# Point-of-Care Ultrasound Pulse Checks During Cardiopulmonary Resuscitation on a Patient Simulator (PUPRAS)

**DOI:** 10.3390/diagnostics15070858

**Published:** 2025-03-27

**Authors:** Susanne Betz, Harald Bergmann, Franz Rettich, Julian Kreutz, Birgit Ploeger, Christoph W. Jaenig, Stephan Grosch, Karl M. Meggiolaro, Andreas Jerrentrup, Willi Schmidbauer, Bernhard Schieffer, Tobias Gruebl

**Affiliations:** 1 Centre for Emergency Medicine, University Hospital Giessen and Marburg, 35043 Marburg, Germany; 2 Department of Anesthesiology, Intensive Care Medicine, Emergency Medicine and Pain Therapy, Bundeswehr Central Hospital, 56072 Koblenz, Germany; 3 Department of Cardiology, Angiology and Medical Intensive Care, University Hospital Giessen and Marburg, 35043 Marburg, Germany; 4 German Red Cross Emergency Medical Service of Mittelhessen gGmbH, Am Krekel 41, 35039 Marburg, Germany; 5 Department of Anesthesiology and Critical Care, University Hospital Giessen and Marburg, 35043 Marburg, Germany

**Keywords:** resuscitation, cardiac arrest, ultrasound, sonography, pulse

## Abstract

**Background/Objectives**: During cardiopulmonary resuscitation (CPR), patients must be checked for signs of return of spontaneous circulation (ROSC). Point-of-care ultrasound (POCUS) may be more reliable for detecting the ROSC. We investigated whether a POCUS pulse check algorithm could be used in compliance with the CPR guidelines. **Methods**: This was a prospective controlled and blinded multicentre manikin study involving staff from two tertiary clinical centres and their emergency medical services. A standard operating procedure for POCUS pulse checks during CPR was evaluated using a simulator in a team of four. The POCUS pulse checks were performed at the central artery following basic and advanced life support. The first pulse check was performed in the setting of pulseless electrical activity, and the second was performed in the presence of ROSC. The participants also completed a questionnaire. **Results**: A total of 444 pulse checks (244 manual/200 POCUS) were performed in 100 scenarios. The participants comprised physicians (34%), nurses (15%), non-physician emergency medical services personnel (37%), and other medical personnel (14%). The pulse checks took an average of 6.7 s (SD 3.9 s). Manual pulse checks (7.3 s) took longer than ultrasound pulse checks (6.1 s; *p* < 0.01), which were performed after a mean of 7.1 min (SD 1.7 min), during the fourth rhythm analysis in 93% of cases, and at the femoral artery in 62% of cases. They were rated as “easy” to perform by 77% and “useful” by 94%. **Conclusions**: POCUS pulse checks basically seem easy to implement and appear to be feasible during CPR.

## 1. Introduction

Sudden cardiac arrest continues to be one of the leading causes of death in industrial nations and is, with an incidence of around 100 per 100,000 inhabitants per year, a fairly common occurrence [1,2,3]. Cardiac arrest is treated through cardiopulmonary resuscitation and follows clearly defined guidelines. The minimal circulation must first be established using external measures, and heart rhythm must be normalised. Various circulation-supporting drugs are used, and the airways must be secured. The main aim is to achieve the return of spontaneous circulation (ROSC) in the patient by treating the cause of the cardiac arrest as best as possible. The proportion of cardiopulmonary resuscitation (CPR) patients with non-shockable rhythms is increasing, as is the number of non-cardiovascular conditions that may be the cause of cardiac arrest [4,5].

Pulse checks provide important guidance for healthcare professionals on when to initiate and especially on when to continue cardiopulmonary resuscitation [6]. Manual palpation for pulse detection, however, has been found to be insufficiently valid [7,8,9,10,11,12]. There are various reasons for this, such as low pulse pressure, agitated medical personnel, or incorrect pulse palpation locations, for example.

Especially in the setting of pulseless electrical activity (PEA), perfusion may be present, but it may not be possible for healthcare professionals to adequately detect a pulse on manual palpation. It therefore makes sense to look for alternative options for more accurate pulse checks during cardiopulmonary resuscitation.

In recent years, point-of-care ultrasound (POCUS) has also become an established tool in the assessment of patients undergoing resuscitation [13,14,15,16,17,18,19,20] and is most commonly used to identify potentially reversible causes of cardiac arrest, to evaluate myocardial movement as a prognostic marker, and to confirm the correct hand placement during chest compressions [21,22,23,24,25,26,27,28,29,30,31,32]. The validity of this measure strongly depends on the skill of the examiner. This skill must therefore be well developed through training. Its integration within cardiopulmonary resuscitation is particularly challenging and requires special intensive training. Only a few studies thus far have investigated the use of ultrasound for the assessment of circulation. They reported that this technique was easy to implement [33,34,35,36]. In particular, it has not been described how and when a pulse check using ultrasound could be integrated into the algorithm. However, astute integration of this examination method into the known resuscitation algorithm is crucial in order to avoid unnecessary pauses and no-flow time.

If the return of spontaneous circulation can be achieved, the resuscitation algorithm is discontinued, and the patient management depends on their treatment needs. Chest compressions that are no longer required then do no further harm to the patient. However, point-of-care ultrasound can then be used to identify the exact causes of the decrease in cardiac output which is often seen in this setting [37,38,39,40,41,42]. To date, no studies have investigated whether and exactly how ultrasound pulse checks can be performed in compliance with the relevant guidelines. This study investigates the precise integration of ultrasound pulse checks on a patient simulator according to a newly developed resuscitation algorithm.

## 2. Materials and Methods

**Figure 1:**
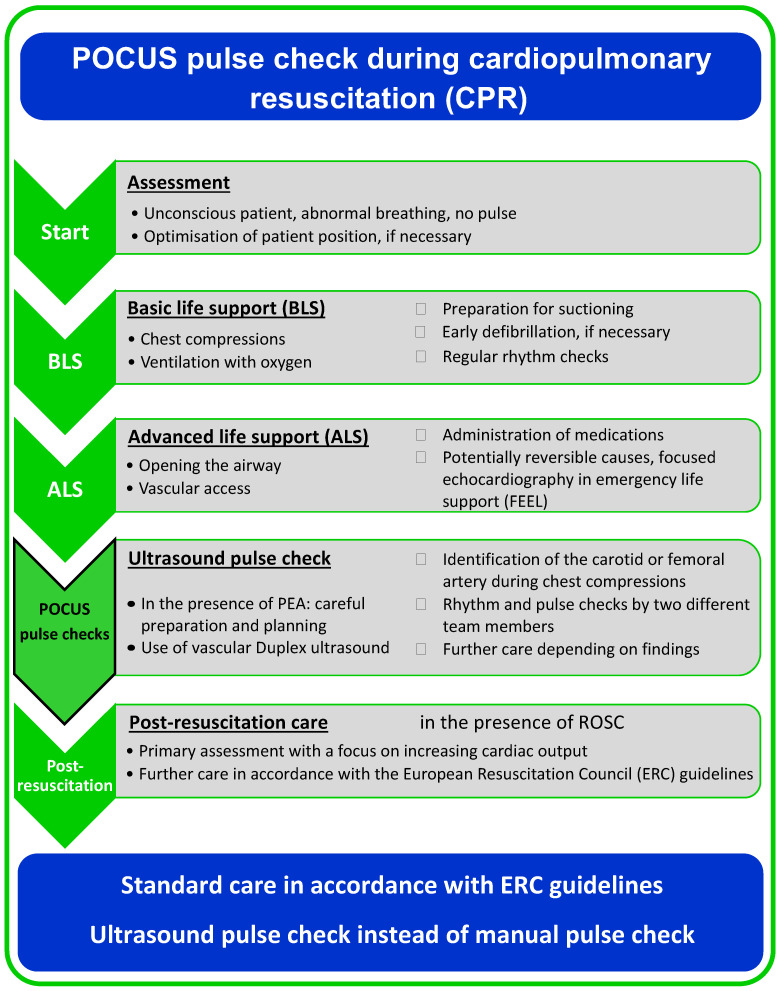
Standard operating procedure for ultrasound pulse checks during cardiopulmonary resuscitation.

After ethical approval was obtained and this study was registered, a standard operating procedure (SOP) for the use of duplex ultrasound for pulse checks during cardiopulmonary resuscitation was evaluated using a patient simulator (Code Blue^®^ III Adult, Gaumard Scientific, Miami, FL, USA) and simulation software (Awesome Ultrasound Simulator, Version 3.6.1, Per Ostergren, Karlstad, Sweden) (Figure 1) in September and October 2021. Priority was given to not delaying the important processes of basic and advanced life support and to integrating ultrasound pulse checks at a prudential point.

**Figure 2:**
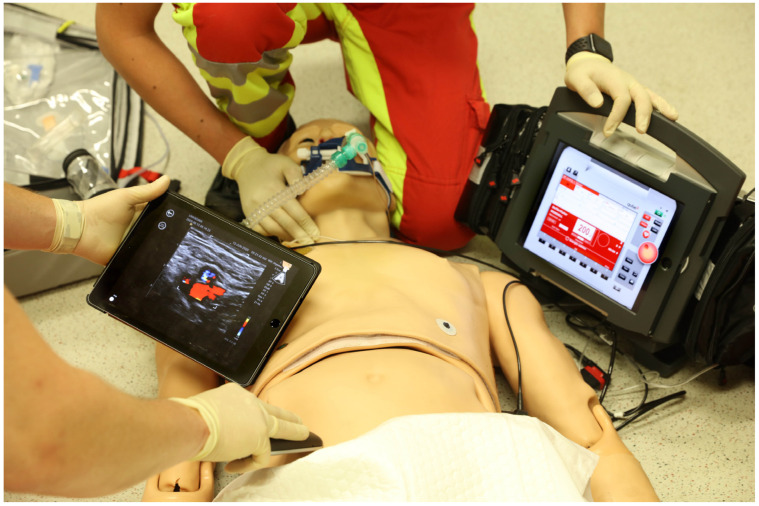
Ultrasound pulse checks at the femoral artery during a rhythm analysis.

According to the standard operating procedure that was used in this study, rhythm and point-of-care ultrasound pulse checks were performed at either the carotid artery or the femoral artery after basic and advanced life support was provided (Figure 2).

In the simulated case scenarios, initial pulse checks and the first pulse check with point-of-care ultrasound were performed in the setting of pulseless electrical activity, and the second pulse check with point-of-care ultrasound was used in the presence of the return of spontaneous circulation in the patient. The only other information provided to the participants was that this was a male patient who was approximately 65 years old and had collapsed unobserved. The participants were presented with preset duplex images. For pulseless electrical activity without the return of spontaneous circulation, no coloured pulse flow and no pulsation of the artery were shown. In the condition of the return of spontaneous circulation, a colour signal and pulsation of the artery could be seen. Every team consisted of four members and included at least one physician as the team leader. In each scenario, the standard equipment for cardiopulmonary resuscitation (an emergency pack with IV lines, infusions, medication, syringes, a bag valve mask, oxygen, a suction unit, a patient monitor with defibrillator, etc.) and also an ultrasound device were available. The participants had not been familiarised with the standardised scenario. However, the new algorithm for integrating ultrasound pulse checks during cardiopulmonary resuscitation was presented before starting the scenario. The teams were formed and dissolved in such a way that direct exchange between the participants was prevented. Team members could participate several times, but the roles of being the team leader and performing the ultrasound pulse check could only be taken on once.

**Figure A1:**
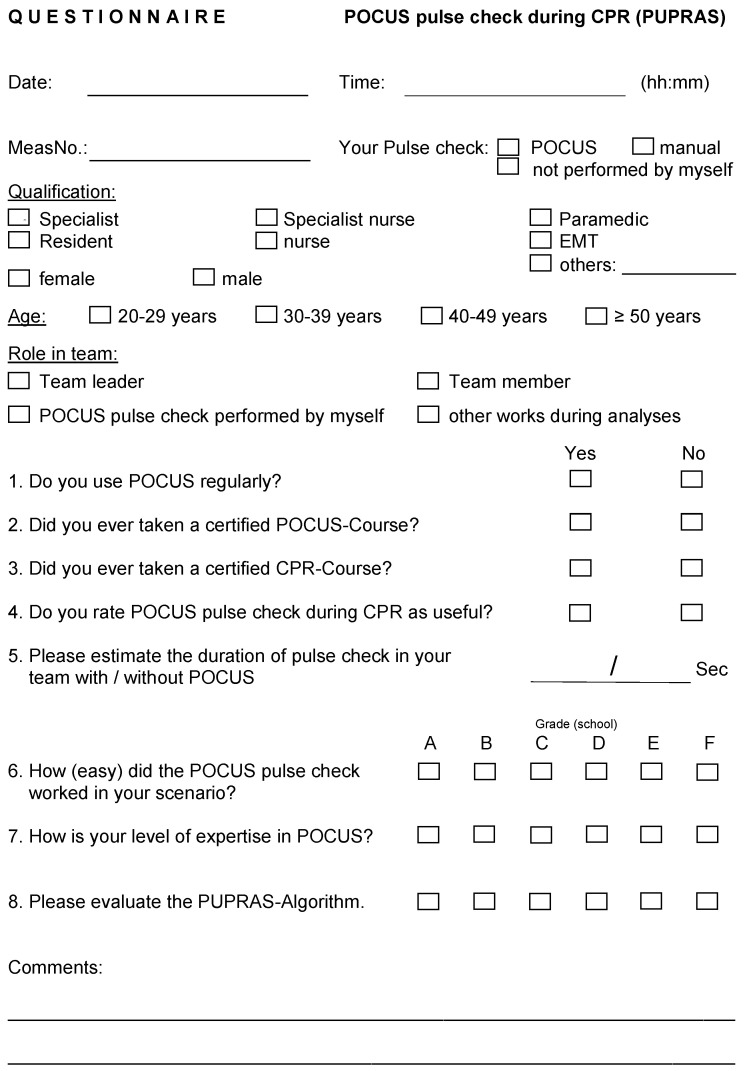
Questionnaire on POCUS pulse checks during CPR (PUPRAS).

During the case scenarios, the time intervals between the beginning of the scenario and relevant time points (the first chest compressions, the first rhythm analysis, first intravenous access, the first administration of vasopressors, airway management, the first rhythm analysis using POCUS, the detection of ROSC) and especially the duration of the pulse checks were recorded. The participants were then asked to evaluate the procedure on the basis of a questionnaire (questionnaire as Appendix A Figure A1 attached).

The primary research question focused on the duration of the point-of-care ultrasound pulse check. The aim was to determine whether a pulse could be measured in less than 10 s using the point-of-care ultrasound method. Based on a random sample, it needed to be shown that the true success rate was significantly greater than 80%. The corresponding null and alternative hypotheses were therefore *H*0 = *π* <= 0.8 and *HA* = *π* > 0.8. Furthermore, it needed to be shown that the point-of-care ultrasound pulse check method did not require significantly more time on average than a classic manual pulse check. A limit of *δ* = 3 s was considered “significant” here. The corresponding null and alternative hypotheses were therefore *H*0 = |*μD*| > 3 and *HA* = |*μD*| <= 3. *μD* denotes the expected difference between the two methods. It was assumed that 90% of the participants would achieve the target of taking an ultrasound pulse measurement in less than 10 s. It was also assumed that the ultrasound pulse check would take the same amount of time (±2 s) as a manual pulse check. A maximum exclusion of 10% of the measurements was assumed. Based on these assumptions, a sample size of at least 408 pulse check measurements was calculated in the power analysis.

Depending on the levels of measurement and the distribution of the variables, medians or means and standard deviations were used as a measure of the central tendency, minimum and maximum values as the extreme values, and quartiles and interquartile ranges as the descriptive measures. The results are presented in box plots. The boxes show the 25% to 75% percentiles with the line in the boxes as the median. The whiskers show the 5% and 95% percentiles. The remaining minima and maxima are shown as dots. Means were compared using Welch’s two one-sided test (TOST) procedure to test the equivalence of the means of two samples (Welch’s *t*-test). Compliance with predetermined time limits was assessed using a generalised estimating equation logit model. Wald’s test was used for significance testing after the null hypotheses were brought to the scale level of the logistic function first. The significance level was set to α = 0.05.

## 3. Results

**Figure 3:**
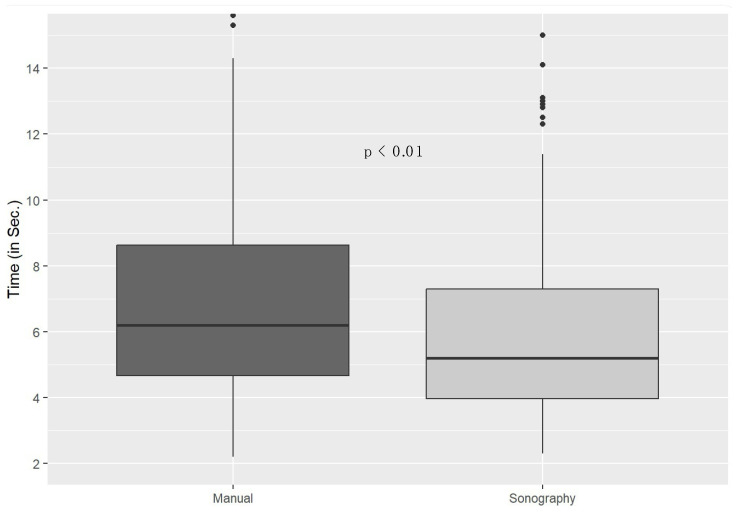
Box plots showing manual and ultrasound pulse check durations.

Following the power analysis, 444 pulse checks (244 manual and 200 point-of-care ultrasound pulse checks), which were performed by 277 participants in 100 case scenarios, were included. The participants comprised physicians (34%; 7% specialists, 27% residents), nurses (15%; 4% specialist nurses in the fields of anaesthesia and intensive care), non-physician emergency medical services (EMS) personnel (37%; 18% paramedics, 19% emergency medical technicians), and other medical personnel, such as emergency care assistants, nursing assistants, interns, and students (14%). The mean age of the participants was 32 years. Of all of the participants, 10% used point-of-care ultrasounds regularly, 5% had taken a certified point-of-care ultrasound course before, and 73% had taken a certified resuscitation course before. The pulse checks took an average of 6.7 s (SD = 3.9 s). Manual pulse checks took significantly longer than point-of-care ultrasound pulse checks (manual checks = 7.3 s; POCUS checks = 6.1 s; *p* < 0.01; r = 0.21; δ = 3 s; Figure 3).

**Figure 4:**
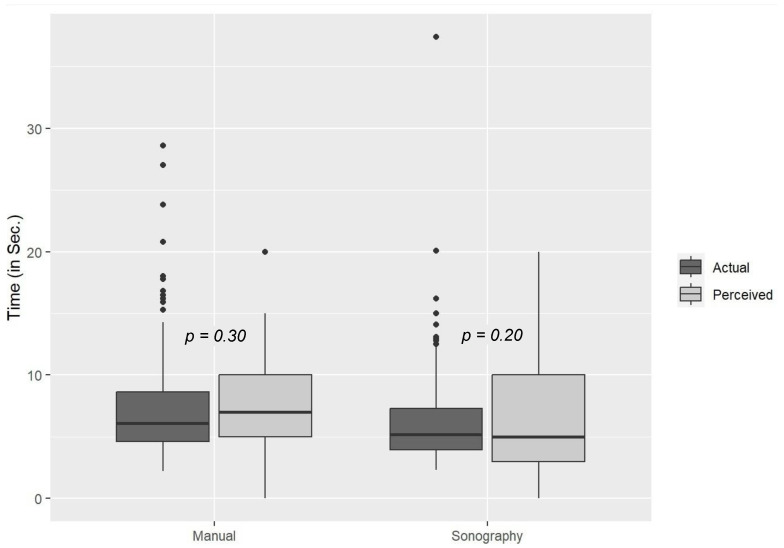
Actual durations of pulse checks versus perceived durations as reported in the questionnaire.

Moreover, 179 out of a total of 200 ultrasound pulse checks took less than 10 s (*p* = 0.0014, π ≤ 0.8). Ultrasound pulse checks were performed after a mean of 7.1 min (SD = 1.7 min), during the fourth rhythm analysis in 93% of cases, and at the femoral artery in 62% of cases. There was no incorrect interpretation of the pulse status. A comparison of these results with the data provided in the questionnaire suggests that the participants had the impression that the duration of the ultrasound pulse checks was longer than that of the manual checks (Figure 4). The difference, however, was not significant.

**Figure 5:**
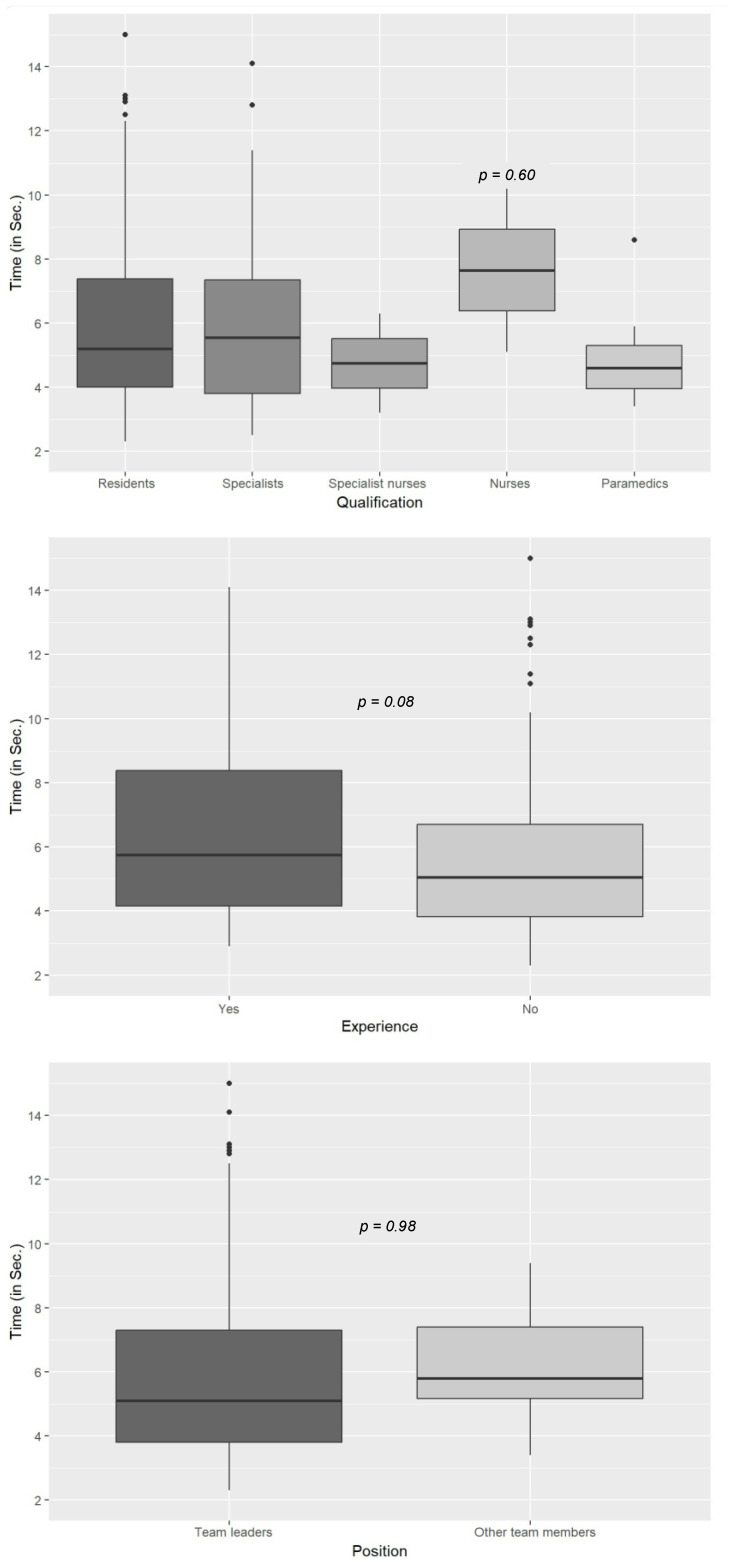
Duration of POCUS pulse checks performed by participants with different professional backgrounds, experience, and roles.

There were no significant differences in the duration of the ultrasound pulse checks between participants from different age groups, of different genders, with different qualifications, or with different levels of ultrasound experience. Likewise, there was no significant difference in the ultrasound pulse check duration between team leaders and other team members (Figure 5). Interestingly, there was a trend of more experienced ultrasound users spending slightly more time on ultrasound pulse monitoring than less experienced users, although this difference was not significant.

No outliers were identified in the data obtained for all participants in terms of the time required to provide basic and advanced life support, such as the time to the first chest compressions, the first rhythm analysis, the first administration of vasopressors, or airway management (Table 1).

The participants complied with the treatment processes and the time intervals defined in cardiopulmonary resuscitation guidelines. In half of the scenarios, a supraglottic device was used for airway management.

Point-of-care ultrasound pulse checks during cardiopulmonary resuscitation were rated as “very easy” or “easy” to perform by 77% and as “useful” during cardiopulmonary resuscitation by 94% of the participants.

## 4. Discussion

Using a predefined standard operating procedure, this study showed that ultrasound pulse checks could be performed by various participants during cardiopulmonary resuscitation on a patient simulator in compliance with the relevant guidelines. The ultrasound pulse checks took less than 10 s on average and did not take longer than the classic manual pulse checks. The scientific study sample analysed reflects the realistic composition of a normal rescue team today in terms of the basic data of the participants, such as their occupational groups or age, for example. The participants first had to provide basic and advanced life support before they performed the ultrasound pulse checks as an adjunct. This means that measures that were decisive for the prognosis (chest compression, ventilation, secured airways, the administration of medication, etc.) were taken first, and compliance with the current recommendations was ensured [4,6]. The present study demonstrated that the applicable algorithms could be used rapidly and pulse checks could be performed quickly using the ultrasound device. Ultrasound pulse checks were found to take even less time than the manual pulse checks on the patient simulator. There was no significant difference in their use in different professional groups or at different experience levels in the ultrasound examination. This is striking because paramedics and nurses use point-of-care ultrasound much less frequently in their daily work than physicians do. The study design allowed the participants to perform the procedures in an ideal setting under identical initial conditions in terms of the simulator, room, and ultrasound images. In this way, the individual measures of the different pulse check methods could be investigated better as they occur in the field using frequently changing situations. This would reflect the decisive difference in the clinical measurements in real patients on the scene. In real-life situations, however, medical personnel may need more time to perform various examinations or treatment measures [36]. In particular, the use of a simulator does not realistically reflect how difficult it can be to detect the vascular structures and obtain ultrasound images in real patients. The first step is to find the correct location of the patient’s vessels. Then, the ultrasound probe should also be positioned centrally and orthogonally to the vessels for an exact analysis. Furthermore, the decision on which ultrasound probe and preset to use must be made. Using normal imaging with a linear probe to detect only possible pulsation of the artery could result in a false negative. In duplex imaging, the appropriate window may first need to be placed on the artery at the correct depth to analyse the target. In addition, interpretation of the image may require more time in the presence of a bradycardic rhythm or a minimal ejection fraction. This inherently requires a longer pulse check time, regardless of whether an ultrasound or a classic manual pulse check is used. However, a recent study by Vojnar et al. showed that point-of-care ultrasound pulse checks could be learned easily in principle [43]. When ultrasound pulse checks are performed by different teams in an ideal setting under identical conditions, the utility and practical implementation of this method can, however, be assessed in a reliable manner. Although simulation-based research provides controlled and replicable circumstances, the results may not completely apply to clinical environments for additional reasons. In actual CPR situations, elements including patient unpredictability, operator stress, and logistical limitations may affect the efficacy of POCUS. The absence of significant differences between the participants from different age groups, from different professional backgrounds, with different qualifications, and with different levels of experience may suggest again that the use of ultrasound for well-defined minor procedures such as pulse checks is intuitive and does not require extensive training. Nevertheless, the success of such applications above all depends on intensive training that focusses on integrating such a procedure into the overall process. It is very important that cardiopulmonary resuscitation is performed using continuous chest compressions in order to maintain the minimal circulation in the patient as well as possible without interruptions. The ultrasound pulse checks that were performed by experienced sonographers tended to take slightly longer. These participants may have attempted not only to detect the presence or absence of a pulse but also to obtain more information from the ultrasound examination. This shows how important a clear focus on the main aim can be, and this is why even experienced users need to be trained in using new procedures in cardiopulmonary resuscitation. Veld et al. described an increased duration of pulse checks when using point-of-care ultrasound in 2017 [44]. However, in the same year, Clattenburg et al. showed faster assessments using point-of-care ultrasound in trained emergency room staff [45]. The ultrasound pulse checks performed by nurses took slightly longer in our study as well. This may be attributable to the fact that nurses are less commonly confronted with patients in cardiac arrest or trained in cardiopulmonary resuscitation less frequently than emergency medical services personnel, for example. This should be considered in the allocation of different tasks to a rescue team during cardiopulmonary resuscitation. Overall, however, this study also showed a trend of the participants having the impression that the duration of ultrasound pulse checks was longer than it actually was. This may have been due to the fact that using a device generally seems more time-consuming than using one’s own fingers or hands. The potentially longer time between starting a device and using it may also increase the perception of the duration of its use. At least, there was no significant difference in the comparison of the objective time measurements of the point-of-care ultrasound pulse checks and the manual pulse checks.

This research did not evaluate the long-term retention of these skills among the participants either. Given that proficiency in POCUS necessitates practice, it would be advantageous to assess whether the performance remains stable over time in the absence of continuous training. Despite this study including a varied participant pool, the majority possessed prior expertise in resuscitation procedures. Therefore, these results may not be immediately relevant to practitioners with little expertise in the application of ultrasound. Although the POCUS pulse assessments were more expedient than the manual evaluations in this study, their practical incorporation into clinical processes requires more scrutiny.

Following a purely objective assessment of the practical implementation of ultrasound pulse checks, the question of what the benefits are for patients should be addressed. After all, the establishment of new procedures in cardiopulmonary resuscitation only really makes sense if their clear benefits have been proven. First of all, the procedure requires that an additional device be taken to the scene. The costs of its acquisition and maintenance, as well as the costs of consumables for its use, must be considered. Moreover, it requires additional time and personnel since one team member must set up and operate the device. Accordingly, an appropriate number of team members must be available. If false negative results are obtained using manual pulse checks and corrected using the additional ultrasound device, the efficiency of the treatment will be considerably increased. In such a situation, resuscitation efforts, which require considerable personnel and material resources, can be discontinued. The entire team can focus on the management of underlying conditions and the patient’s transport to the nearest appropriate treatment facility. Less effective measures with potential risks, such as rib fractures during chest compressions, e.g., can then be avoided. The use of ultrasound pulse checks can thus have substantial consequences that may lead to better treatment outcomes. The available evidence does not show conclusively whether a pulse that can be detected only via ultrasound is indicative of circulation that is not (yet) sufficient (no palpable pulse) and whether a patient in this condition would benefit more from continued cardiopulmonary resuscitation efforts than from focused attempts at cardiovascular stabilisation. Further chest compressions may maintain better cardiac perfusion and support stabilisation of the circulation until pulsation of the artery is palpable again. This question is particularly relevant in cases of pulseless electrical activity in rhythm checks during cardiopulmonary resuscitation. At least, high-dose boluses of vasopressors should be withheld in these situations, similar to when a patient’s end-tidal carbon dioxide increases during cardiopulmonary resuscitation [4,46].

However, these results only show what happens on a patient simulator. Other circumstances may arise in real patients undergoing cardiac arrest and in practical environments. That is why this study is only a first step in establishing new methods of examination during cardiopulmonary resuscitation. Large-scale randomised controlled trials should be performed in patients in order to assess the actual effects and benefits before this procedure is widely used in practice.

## 5. Conclusions

In this study, even inexperienced sonographers were able to perform point-of-care ultrasound pulse checks at the central artery on a patient simulator during cardiopulmonary resuscitation following a special and new algorithm in compliance with the relevant guidelines. Members of the cardiopulmonary resuscitation teams rated this procedure as easy to perform and useful. Some limitations of this study do not yet allow for the direct application of this treatment to real patients because many factors could influence the results and the effects of this special measure. Clinical studies should be conducted in order to assess the benefits of this tool for patients in cardiac arrest.

## Figures and Tables

**Table 1:** Mean time intervals between the beginning of the scenario and relevant time points.

	Time Interval
Time to first chest compression	17 (±8) s
Time to first rhythm analysis	1.7 (±0.8) min
Time to intravenous access	2.9 (±1.0) min
Time to first administration of vasopressors	3.7 (±1.3) min
Time to airway management	4.1 (±1.8) min
	[ETT = 4.1 (±1.6) min; SGA = 4.0 (±2.1) min]
Time to first POCUS pulse check	7.1 (±1.7) min
Time to detection of ROSC	8.9 (±1.8) min

ETT—endotracheal tube; SGA—supraglottic airway; POCUS—point-of-care ultrasound; ROSC—return of spontaneous circulation; s—seconds; min—minutes.

## Data Availability

The data underlying the results presented in this paper are not publicly available at this time but may be obtained from the authors upon reasonable request.

## Abbreviations

The following abbreviations are used in this manuscript:

ALS	Advanced life support
BLS	Basic life support
CPR	Cardiopulmonary resuscitation
EMS	Emergency medical services
ERC	European Resuscitation Council
ETT	Endotracheal tube
FEEL	Focused echocardiography in emergency life support
PEA	Pulseless electrical activity
POCUS	Point-of-care ultrasound
PUPRAS	Point-of-care ultrasound pulse checks during cardiopulmonary resuscitation on patient simulator
ROSC	Return of spontaneous circulation
SD	Standard deviation
SGA	Supraglottic airway
SOP	Standard operating procedure
TOST	Two one-sided test
